# Supplementary material for: School-Age Outcomes of Antenatal Magnesium Sulphate in Preterm Infants
Source: Children (Basel). 2023 Jul 31;10(8):1324. doi: 10.3390/children10081324 (PMC10453514; doi:10.3390/children10081324)
Supplement: Supplementary file 1 [file children-10-01324-s001.zip › Supporting information S1.pdf]

Search Strategies

A. EMBASE

1 magnesium sulfate/

2 magnesium sulphate.tw.

3 or/1-2

4 prenatal care/

5 (antenatal or "ante natal" or prenatal or "pre natal" or preterm).tw.

6 threatened premature delivery.tw.

7 or/4-6

8 (clin$ adj2 trial).mp.

9 ((singl$ or doubl$ or trebl$ or tripl$) adj (blind$ or mask$)).mp.

10 (random$ adj5 (assign$ or allocat$)).mp.

11 randomi$.mp.

12 crossover.mp.

13 exp randomized-controlled-trial/

14 exp double-blind-procedure/

15 exp crossover-procedure/

16 exp single-blind-procedure/

17 exp randomization/

18 or/8-17

19 3 and 7 and 18

20 limit 19 to yr="2008 -Current"

B. MEDLINE

1 Magnesium Sulfate/

2 magnesium sulphate.tw.

3 or/1-2

4 Prenatal Care/

5 (antenatal or "ante natal" or prenatal or "pre natal" or preterm).tw.

6 threatened premature delivery.tw.

7 or/4-6

8 exp clinical trial/

9 exp randomized controlled trials/

10 exp double-blind method/

11 exp single-blind method/

12 exp cross-over studies/

13 randomized controlled trial.pt.

14 clinical trial.pt.

15 controlled clinical trial.pt.

16 (clinic$ adj2 trial).mp.

17 (random$ adj5 control$ adj5 trial$).mp.

18 (crossover or cross-over).mp.

19 ((singl$ or double$ or trebl$ or tripl$) adj (blind$ or mask$)).mp.

20 randomi$.mp.

21 (random$ adj5 (assign$ or allocat$ or assort$ or reciev$)).mp.

22 or/8-21

23 3 and 7 and 22

24 limit 23 to yr="2008 -Current"

C. CINAHL

S21 S12 AND S15 AND S19

S20 S12 AND S15 AND S19

S19 S16 OR S17 OR S18

S18 TX threatened premature delivery

S17 TX (antenatal or "ante natal" or prenatal or "pre natal" or preterm)

S16 (MH "Prenatal Care")

S15 S13 OR S14

S14 TX magnesium sulphate

S13 (MH "Magnesium Sulfate")

S12 S1 OR S2 OR S3 OR S4 OR S5 OR S6 OR S7 OR S8 OR S9 OR S10 OR S11

S11 TX allocat* random*

S10 (MH "Quantitative Studies")

S9 (MH "Placebos")

S8 TX placebo*

S7 TX random* allocat*

S6 (MH "Random Assignment")

S5 TX randomi* control* trial*

S4 TX ((singl* n1 blind*) or (singl* n1 mask*)) or TX ((doubl* n1 blind*) or (doubl* n1 mask*)) or TX ((tripl* n1 blind*) or (tripl* n1 mask*)) or TX ((trebl* n1 blind*) or (trebl* n1 mask*))

S3 TX clinic* n1 trial*

S2 PT Clinical trial

S1 (MH "Clinical Trials+")

D. Cochrane

#1 MeSH descriptor: [Magnesium Sulfate] this term only

#2 magnesium sulphate:ti,ab,kw (Word variations have been searched)

#3 #1 or #2 [2699]

#4 MeSH descriptor: [Prenatal Care] this term only

#5 (antenatal or "ante natal" or prenatal or "pre natal" or preterm):ti,ab,kw (Word variations have been searched)

#6 #4 or #5

#7 #3 and #6 Publication Year from 2008 to 2020, in Trials
