# Supplementary material for: School-Age Outcomes of Antenatal Magnesium Sulphate in Preterm Infants
Source: Children (Basel). 2023 Jul 31;10(8):1324. doi: 10.3390/children10081324 (PMC10453514; doi:10.3390/children10081324)
Supplement: Supplementary file 1 [file children-10-01324-s001.zip › Supporting information S2 and S4.pptx]

## Slide 1
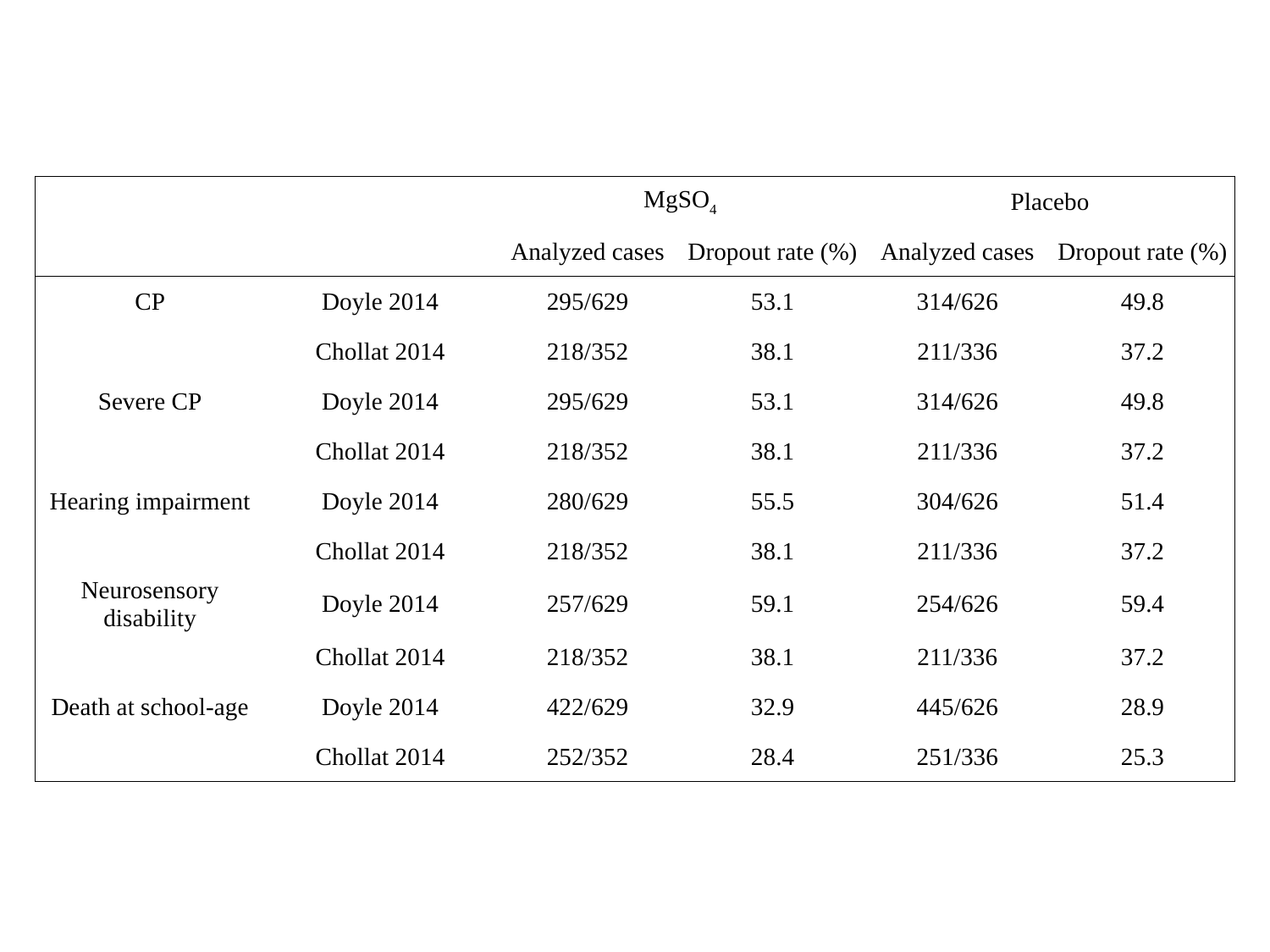

| | | MgSO4 | | Placebo | |
| --- | --- | --- | --- | --- | --- |
| | | Analyzed cases | Dropout rate (%) | Analyzed cases | Dropout rate (%) |
| CP | Doyle 2014 | 295/629 | 53.1 | 314/626 | 49.8 |
| | Chollat 2014 | 218/352 | 38.1 | 211/336 | 37.2 |
| Severe CP | Doyle 2014 | 295/629 | 53.1 | 314/626 | 49.8 |
| | Chollat 2014 | 218/352 | 38.1 | 211/336 | 37.2 |
| Hearing impairment | Doyle 2014 | 280/629 | 55.5 | 304/626 | 51.4 |
| | Chollat 2014 | 218/352 | 38.1 | 211/336 | 37.2 |
| Neurosensory disability | Doyle 2014 | 257/629 | 59.1 | 254/626 | 59.4 |
| | Chollat 2014 | 218/352 | 38.1 | 211/336 | 37.2 |
| Death at school-age | Doyle 2014 | 422/629 | 32.9 | 445/626 | 28.9 |
| | Chollat 2014 | 252/352 | 28.4 | 251/336 | 25.3 |

## Slide 2
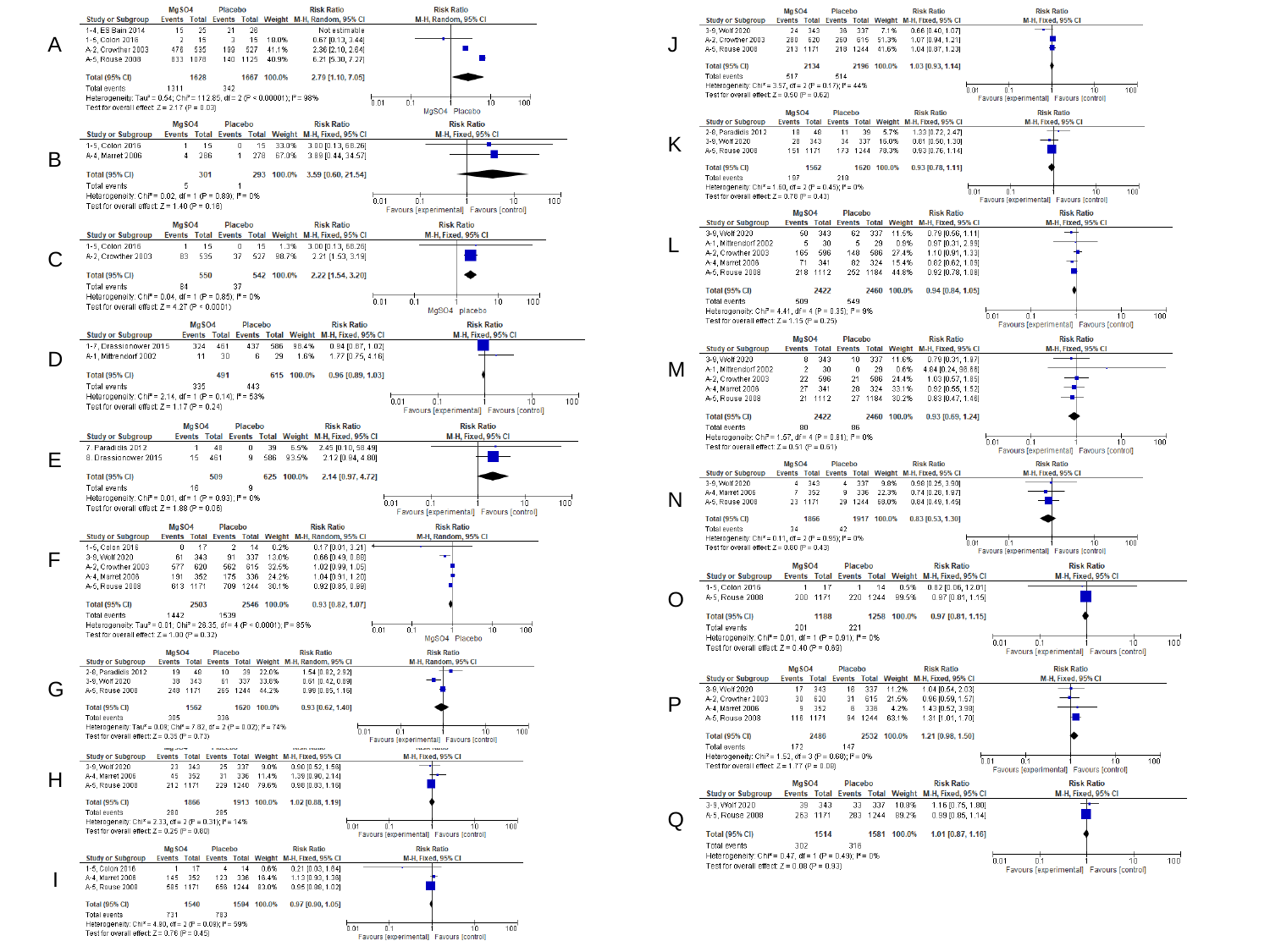

A
B
C
D
E
F
G
H
I
J
K
L
M
N
O
P
Q
