# Supplementary material for: School-Age Outcomes of Antenatal Magnesium Sulphate in Preterm Infants
Source: Children (Basel). 2023 Jul 31;10(8):1324. doi: 10.3390/children10081324 (PMC10453514; doi:10.3390/children10081324)
Supplement: Supplementary file 1 [file children-10-01324-s001.zip › Supporting information S3.pptx]

## Slide 1
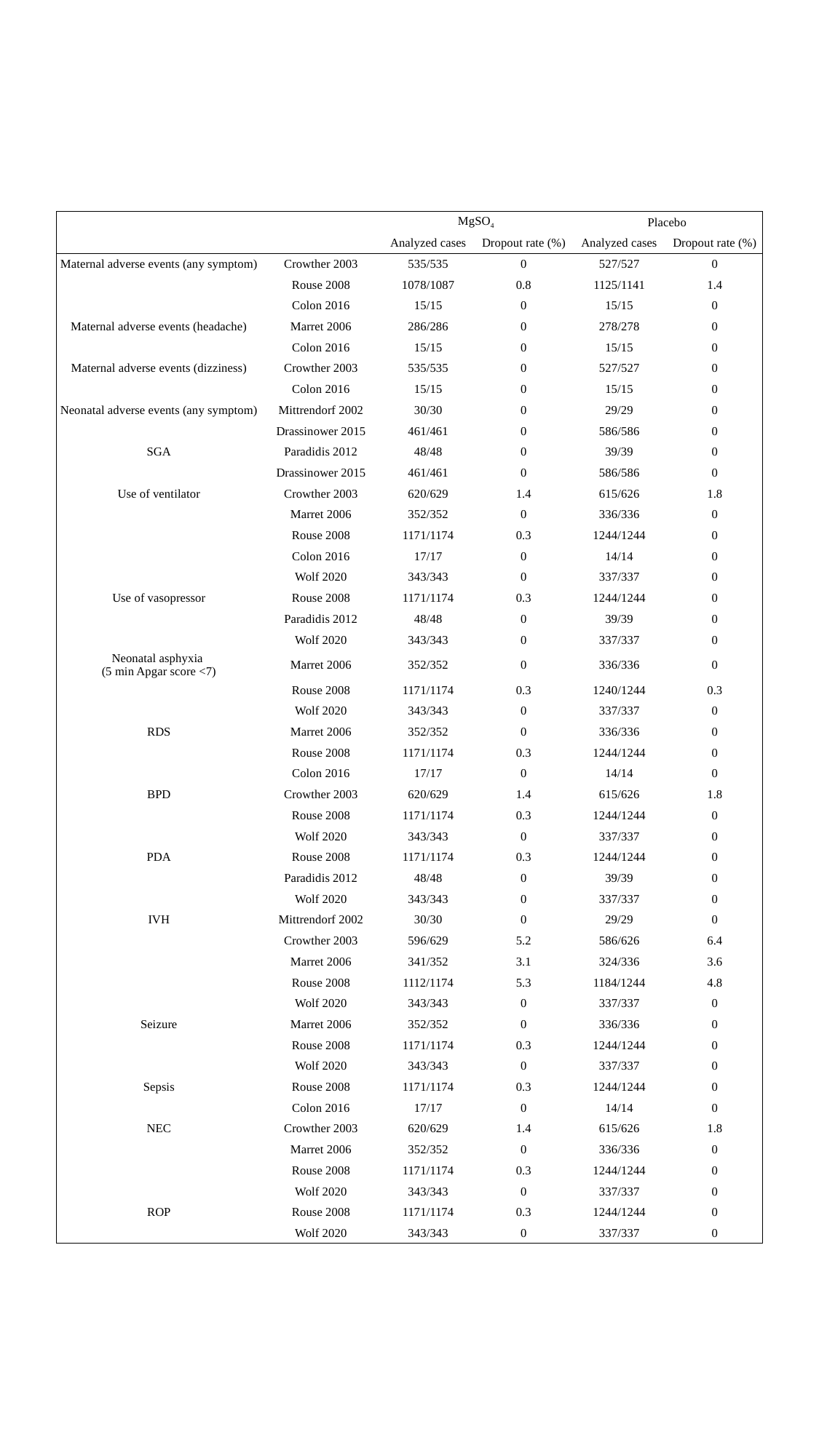

| | | MgSO4 | | Placebo | |
| --- | --- | --- | --- | --- | --- |
| | | Analyzed cases | Dropout rate (%) | Analyzed cases | Dropout rate (%) |
| Maternal adverse events (any symptom) | Crowther 2003 | 535/535 | 0 | 527/527 | 0 |
| | Rouse 2008 | 1078/1087 | 0.8 | 1125/1141 | 1.4 |
| | Colon 2016 | 15/15 | 0 | 15/15 | 0 |
| Maternal adverse events (headache) | Marret 2006 | 286/286 | 0 | 278/278 | 0 |
| | Colon 2016 | 15/15 | 0 | 15/15 | 0 |
| Maternal adverse events (dizziness) | Crowther 2003 | 535/535 | 0 | 527/527 | 0 |
| | Colon 2016 | 15/15 | 0 | 15/15 | 0 |
| Neonatal adverse events (any symptom) | Mittrendorf 2002 | 30/30 | 0 | 29/29 | 0 |
| | Drassinower 2015 | 461/461 | 0 | 586/586 | 0 |
| SGA | Paradidis 2012 | 48/48 | 0 | 39/39 | 0 |
| | Drassinower 2015 | 461/461 | 0 | 586/586 | 0 |
| Use of ventilator | Crowther 2003 | 620/629 | 1.4 | 615/626 | 1.8 |
| | Marret 2006 | 352/352 | 0 | 336/336 | 0 |
| | Rouse 2008 | 1171/1174 | 0.3 | 1244/1244 | 0 |
| | Colon 2016 | 17/17 | 0 | 14/14 | 0 |
| | Wolf 2020 | 343/343 | 0 | 337/337 | 0 |
| Use of vasopressor | Rouse 2008 | 1171/1174 | 0.3 | 1244/1244 | 0 |
| | Paradidis 2012 | 48/48 | 0 | 39/39 | 0 |
| | Wolf 2020 | 343/343 | 0 | 337/337 | 0 |
| Neonatal asphyxia (5 min Apgar score <7) | Marret 2006 | 352/352 | 0 | 336/336 | 0 |
| | Rouse 2008 | 1171/1174 | 0.3 | 1240/1244 | 0.3 |
| | Wolf 2020 | 343/343 | 0 | 337/337 | 0 |
| RDS | Marret 2006 | 352/352 | 0 | 336/336 | 0 |
| | Rouse 2008 | 1171/1174 | 0.3 | 1244/1244 | 0 |
| | Colon 2016 | 17/17 | 0 | 14/14 | 0 |
| BPD | Crowther 2003 | 620/629 | 1.4 | 615/626 | 1.8 |
| | Rouse 2008 | 1171/1174 | 0.3 | 1244/1244 | 0 |
| | Wolf 2020 | 343/343 | 0 | 337/337 | 0 |
| PDA | Rouse 2008 | 1171/1174 | 0.3 | 1244/1244 | 0 |
| | Paradidis 2012 | 48/48 | 0 | 39/39 | 0 |
| | Wolf 2020 | 343/343 | 0 | 337/337 | 0 |
| IVH | Mittrendorf 2002 | 30/30 | 0 | 29/29 | 0 |
| | Crowther 2003 | 596/629 | 5.2 | 586/626 | 6.4 |
| | Marret 2006 | 341/352 | 3.1 | 324/336 | 3.6 |
| | Rouse 2008 | 1112/1174 | 5.3 | 1184/1244 | 4.8 |
| | Wolf 2020 | 343/343 | 0 | 337/337 | 0 |
| Seizure | Marret 2006 | 352/352 | 0 | 336/336 | 0 |
| | Rouse 2008 | 1171/1174 | 0.3 | 1244/1244 | 0 |
| | Wolf 2020 | 343/343 | 0 | 337/337 | 0 |
| Sepsis | Rouse 2008 | 1171/1174 | 0.3 | 1244/1244 | 0 |
| | Colon 2016 | 17/17 | 0 | 14/14 | 0 |
| NEC | Crowther 2003 | 620/629 | 1.4 | 615/626 | 1.8 |
| | Marret 2006 | 352/352 | 0 | 336/336 | 0 |
| | Rouse 2008 | 1171/1174 | 0.3 | 1244/1244 | 0 |
| | Wolf 2020 | 343/343 | 0 | 337/337 | 0 |
| ROP | Rouse 2008 | 1171/1174 | 0.3 | 1244/1244 | 0 |
| | Wolf 2020 | 343/343 | 0 | 337/337 | 0 |
